# Pediatric Subcutaneous Abscess: Still a Clinical Exam-Based Diagnosis and Treatment

**DOI:** 10.3390/children8050392

**Published:** 2021-05-14

**Authors:** Isabel C. Garcia, Rachael A. Clark, Dai H. Chung, Nakia Gaines

**Affiliations:** 1 Department of Surgery, University of Texas Southwestern Medical Center, Dallas, TX 75390, USA; IsabelC.Garcia@UTSouthwestern.edu (I.C.G.); rachael.clark@utsouthwestern.edu (R.A.C.); 2 Department of Pediatrics, University of Texas Southwestern Medical Center, Dallas, TX 75390, USA; Nakia.Gaines@UTSouthwestern.edu

**Keywords:** subcutaneous, abscess, management

## Abstract

Subcutaneous abscesses occur frequently in the pediatric population, yet there is great variability in the approach to diagnosis and management, partly due to opposing recommendations in the current literature and the lack of a standardized protocol for diagnosis and management among pediatric medical centers. This has led to inconsistencies by the providers, as well as the hospital clinical pathways, with regards to the appropriate management of subcutaneous abscesses. We hypothesize that the current variability in diagnostic work-up and management contributes to the wide use of unnecessary imaging and therapeutics without altering the overall outcomes. We performed a retrospective chart review that compared 200 encounters for patients < 18 years of age with a diagnosis of subcutaneous abscess at a single large tertiary pediatric institution. Our results showed that only 13.6% of wound cultures obtained led to a change in the antibiotic regimen and that blood cultures were positive in only 2.1% of cases. There was no difference in the incision and drainage performed based on ultrasound findings in the presence of fluctuance on physical exam. Patients presenting with fever were more likely to be admitted to the hospital for further care than those without fever. Our results showed no difference in outcome after incision and drainage for abscesses packed with gauze versus those left to drain via a vessel loop drain. There was no difference in recurrence in patients discharged with oral antibiotics versus without oral antibiotic treatment. Our data indicate that many of the diagnostic studies used for the management of a subcutaneous abscess have little to no effect on the outcome. Subcutaneous abscesses are a common pediatric complaint, and this study could help healthcare providers utilize more effective and efficient management strategies for skin and soft tissue infections.

## 1. Introduction

Skin and soft-tissue infections (SSTIs) are a relatively common diagnosis in both the pediatric and adult populations and include abscesses and cellulitis. An abscess is a focal, contained, purulent infection with a clearly defined “cavity” and surrounding inflammation involving the deep subcutaneous tissues. Cellulitis is an infection of the skin, without an organized cavity, presenting with erythema, warmth, induration, and tenderness [1]. There has been a recent rise in the incidence of SSTIs, with up to 95% of this increase attributed to abscesses and cellulitis, with the largest change occurring in the pediatric (<18 years) population [2]. In addition to an overall increase in SSTIs, there has been a concomitant rise in the incidence of community-acquired methicillin-resistant *Staphylococcus aureus* (CA-MRSA) [3]. The increase in cases has led to an increased variation in practice in the diagnosis and management of subcutaneous abscesses, particularly in the pediatric population. The routine use of laboratory testing, diagnostic imaging, wound cultures, and antibiotic management are common areas of debate.

At times, the presence of an abscess can be difficult to discern from cellulitis on clinical examination alone and cellulitis and abscesses are not mutually exclusive. On clinical exam, the presence of fluctuance, which signifies purulent material within a cavity, can correspond with the presence of an abscess [1]. However, fluctuance is not always easy to determine, especially in cases with extensive cellulitis and skin induration. The use of diagnostic ultrasound has become more prevalent, especially with the increasing popularity of point-of-care ultrasound (POCUS). POCUS has been shown to improve diagnostic accuracy in children with SSTIs [4] and is beneficial in cases of clinical uncertainty [5].

The gold standard treatment for subcutaneous abscesses remains incision and drainage (I&D) [6]. The use of antibiotics for SSTIs after drainage is an area of great debate and variance. As a general guideline, the Infectious Disease Society of America (IDSA) recommends the use of antibiotics after I&D only in the presence of systemic signs of infection [6]. In the pediatric population, several studies have shown that there is no significant difference between adjunct antibiotics post-I&D and I&D alone [7,8,9,10]. Obtaining blood cultures has become a common practice in pediatric patients presenting with SSTIs. However, while more than 90% of SSTI patients undergo laboratory investigations, including blood cultures, less than 1% of patients with uncomplicated SSTIs yield a positive blood culture [11]. Furthermore, the IDSA guidelines do not specify the need for blood cultures in healthy, immunocompetent patients, but do suggest blood cultures in neutropenic and immunocompromised patients [6].

The current approach to subcutaneous abscess not only varies across institutions but among practitioners within the same institution. Our institution does not have a standard protocol in place for the diagnosis and management of subcutaneous abscesses. We hypothesize that the current variability in diagnostic procedures and management is leading to the use of unnecessary diagnostic procedures, laboratory evaluation, and antibiotic use. The aim of the current study is to identify and describe the variability in the diagnosis and management of subcutaneous abscesses at a large tertiary children’s hospital in order to develop a streamlined approach to the diagnosis and treatment of subcutaneous abscess in the pediatric population.

## 2. Materials and Methods

### 2.1. Study Design

After approval from the Institutional Review Board (IRB#2020-0065), a single-center, retrospective chart review was performed. All children between the ages of two months and 18 years who were evaluated in the Emergency Department (ED) for a subcutaneous gluteal/buttock SSTI from 1 January 2019 to 31 December 2019 were included. Patients were identified based on ICD-9-CM and ICD-10-CM codes. Immunocompromised patients, patients with complications from prior wounds or surgeries, and those with an incidental finding of abscess were excluded.

### 2.2. Clinical Variables.

Data collected included: demographics, presenting symptoms, past medical history, exam findings, laboratory values, culture results, imaging results, procedures, and outcomes. Presenting symptoms included: symptom onset, duration, location, subjective fever, and prior treatment received, including duration of oral antibiotics, if applicable. Past medical history included: co-morbidities, prior abscesses or recent hospitalization (within 30 days), and history of previous abscesses/SSTIs or MRSA. Exam findings, as documented in clinician notes, included vital signs, surrounding cellulitis and/or fluctuance. Laboratory studies included: White Blood Cell (WBC) count and C-reactive protein (CRP) as well as wound and/or blood cultures. Outcomes included the need for admission, antibiotic administration, length of stay (LOS), discharge medications, and recurrence. Recurrence was defined as the return to the ED or pediatrician for an abscess in the same location or at close proximity within 30 days of hospital discharge.

### 2.3. Statistical Analysis

Qualitative data are reported as *n* (% of total). Quantitative variables are reported as the mean ± standard deviation (SD). Univariate associations of quantitative variables were analyzed using Student’s *t*-tests. Qualitative variables were analyzed by cross-tabulation, Pearson χ^2^, and McNemar tests. Data analysis was performed using IBM SPSS (version 26, 2019, Armonk, NY, USA), and statistical significance was determined at a *p* value < 0.05.

## 3. Results

### 3.1. Demographics and Patient Characteristics

Out of the 212 patients with gluteal SSTIs that were identified, 200 patients met inclusion criteria. Demographic information and patient characteristics are summarized in Table 1. Less than half of all patients were boys (44.0%) with an average age of 4.75 ± 5.4 years. While only 11 (5.5%) patients had co-morbidities, 60 (30%) had a prior history of abscess and 12 (6.0%) had a documented history of previous MRSA infection. Fifty percent of patients reported a subjective fever, and nearly one-third of patients (29%) had been prescribed oral antibiotics prior to presentation with an average duration of 3.97 ± 4.9 days. There was no significant difference in patient characteristics, clinical exam, laboratory findings, management, or long-term outcomes between children treated with oral antibiotics prior to presentation (Supplemental Table S1). Cellulitis was evident on physical exam in 110 (55.0%) patients, whereas fluctuance was recognized in 81 (40.5%) patients. The combination of cellulitis and fluctuance was present in 42 (21.0%) patients. There were 29 patients with multiple abscesses on initial exam. The average vital signs on presentation are summarized in Table 1.

### 3.2. Diagnostic Evaluation

**Figure 1:**
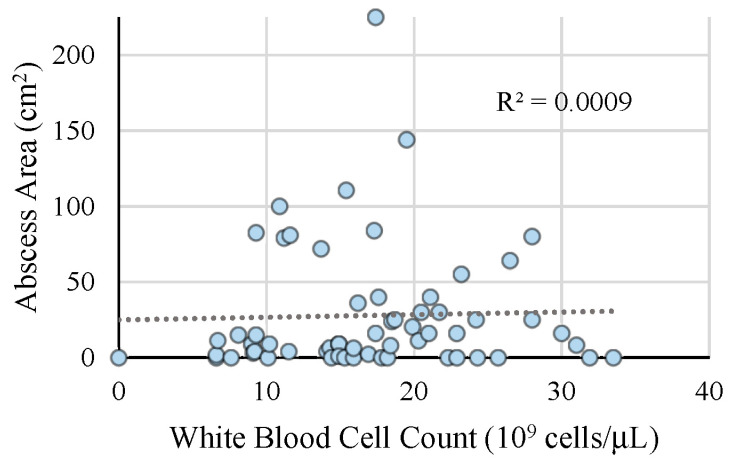
Abscess area correlates with white blood cell count. A scatter plot representing the area of the abscess (cm^2^) corresponding to the white blood cell count (10^9^ cells/µL) with a linear trendline (R^2^ = 0.0009). The area of the abscess was calculated using the dimensions provided by ultrasonography when available. If no dimensions on ultrasonography were available, the area was calculated using the dimensions of induration and erythema, and the abscess was assumed to be the shape of a square when a length and width were provided.

As seen in Table 1, the average WBC count was 17.5 ± 6.9 × 10^9^ cells/L and the average CRP level was 6.4 ± 5.2 mg/L. The size of the abscess area (as measured by ultrasound or physical exam) was measured as 24.4 ± 34.9 cm^2^. Our results showed that there was no correlation between the abscess size and WBC count, R2 = 0.0009 (Figure 1). In addition, as seen in Table 2, there was no significant difference in patients undergoing I&D versus no I&D based on laboratory values such as the WBC count and CRP level. There was also no difference in vital signs including temperature, heart rate, and respiratory rate. The abscess area was larger in the I&D group compared to the no I&D group; however, this was not statistically significant (28.8 ± 39.4 vs. 17.2 ± 24.5, *p* = 0.05).

**Figure 2:**
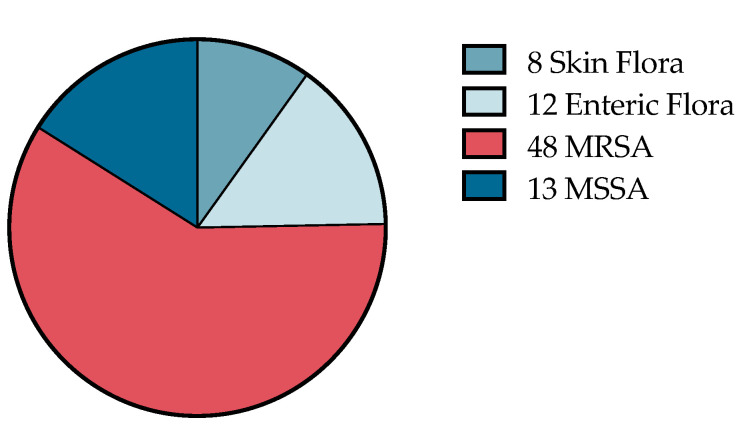
Bacteria identified in abscess wound cultures taken from abscesses. The most common bacteria identified was methicillin-resistant *Staphylococcus aureus* (MRSA) followed by methicillin-sensitive *Staphylococcus aureus.* The remainder showed skin and enteric flora.

Overall, wound and blood cultures were obtained in 41.5% and 21.5% of patients, respectively. However, this led to an antibiotic regimen change in only 13.6% of the wound cultures ordered and 6.0% of the total encounters. A summary of the bacteria identified in the abscesses based on the wound culture results is found in Figure 2. Only one out of 43 blood cultures ordered was positive.

**Figure 3:**
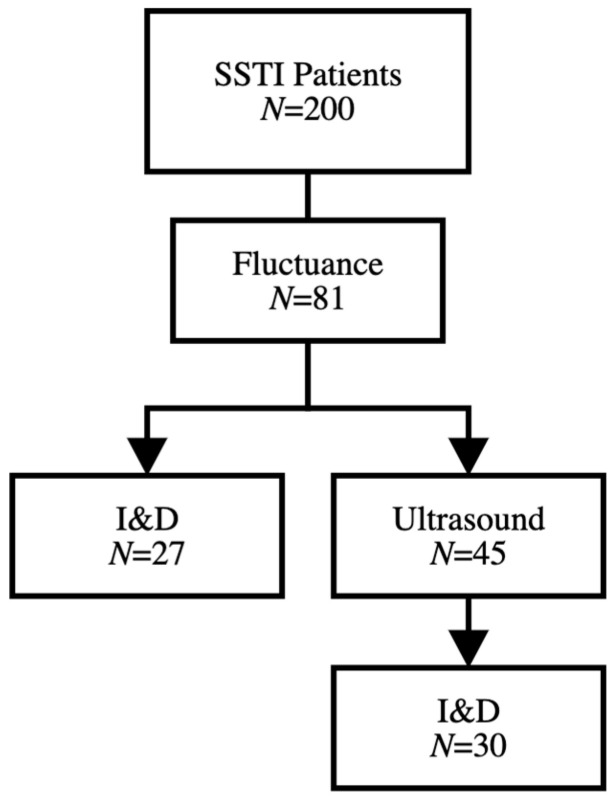
Utility of clinical exam versus ultrasound for detecting operative abscess. Patients presenting with fluctuance on clinical exam underwent incision and drainage directly when compared to those who underwent additional imaging prior in order to confirm the presence of an abscess prior to incision and drainage.

Ultrasound study was performed in 108 (54.0%) patients overall, including 45 patients with documented fluctuance on physical exam, as seen in Figure 3. Children who received oral antibiotics prior to presentation were less likely to undergo ultrasound evaluation compared to children without prior antibiotic treatment (Supplemental Table S1). A total of nine patients with no evidence of cellulitis or fluctuance on exam had evidence of subcutaneous abscess on ultrasound, and only three underwent I&D. Of the 45 patients in which an ultrasound was performed, the diagnosis of abscess was confirmed in 32 cases. Of those 32 cases, 30 (93.8%) patients underwent I&D, while two (6.8%) were treated conservatively with topical EMLA (lidocaine 2.5% and prilocaine 2.5%) cream and sitz baths. Similarly, out of 81 patients that had fluctuance on physical exam, three (3.7%) were treated with topical EMLA, four (4.9%) were spontaneously draining and three (3.7%) had a negative ultrasound. Interestingly, 27 (33.3%) underwent I&D without an ultrasound, suggesting no change in treatment based on ultrasound imaging.

### 3.3. Treatment and Outcomes

An I&D was performed in 121 (60.5%) patients with recurrence in 12.5% of patients. As seen in Table 2, patients with fluctuance on exam were significantly more likely to undergo I&D compared to those without fluctuance (29.0% vs. 11.5%, *p* = 0.005). There was no significant difference between those with cellulitis on exam compared to those without cellulitis (57.9% vs. 50.6%, *p* = 0.07). Among the 108 patients who underwent ultrasound, operative I&D was performed in 65 patients compared to 43 patients who did not require an I&D (53.7 vs. 54.4, *p* = 1.0).

As seen in Table 3, a total of 55 patients were admitted. The age of admitted patients was significantly lower than those who were discharged from the ED (3.4 ± 4.5 vs. 5.3 ± 5.6 years, *p* = 0.02). Out of the 121 I&Ds performed, 37 (67.3%) patients were admitted and 84 (57.9%) were discharged from the ED. While there was no significant difference in temperature between admitted versus discharged patients (37.1 ± 0.7 vs. 37.3 ± 1.0 °C, *p* = 0.14), the heart rates and respiratory rates were significantly higher in admitted patients. There was no significant association between fluctuance and admission (*p* = 0.56); however, the abscess cavity size was significantly larger in admitted patients compared to discharged patients (47.8 ± 50.5 vs. 16.2 ± 22.7 cm^2^, *p* = 0.001). As expected, the WBC count (19.1 ± 6.9 × 10^9^ cells/L) and CRP level (8.0 ± 5.2 mg/L) were significantly higher in admitted patients.

After I&D, the wound was packed with gauze in 36 (18%) patients, and a loop drain was placed in 17 (8.5%) patients. Seventy patients were treated with intravenous (IV) antibiotics. There was no association between the temperature of patients who received vs. those who did not receive IV antibiotics (37.1 ± 0.66 vs. 37.3 ± 0.99, *p* = 0.10). A total of 42 (21.0%) patients were discharged with a course of oral antibiotics. As seen in Table 4, recurrence occurred within 30 days in 16 (8.0%) patients overall and in four (2.0%) patients discharged on oral antibiotics compared to 12 (6.0%) patients without oral antibiotics. However, this difference was not significant (*p* = 0.63). IV antibiotic use was associated with decreased recurrence (5 vs. 11, *p* = 0.005). Neither packing (*p* = 0.29) nor loop drain placement (*p* = 0.09) were associated with a significantly decreased recurrence. There was no difference in recurrence based on the patient temperature, HR, RR, BMI, WBC, CRP, or abscess size.

## 4. Discussion

Despite an increasing prevalence in SSTIs such as subcutaneous abscesses in the pediatric population, a wide variability in the evaluation and management remains. In the present study, we sought to identify and describe the variability in the diagnosis and management of subcutaneous abscesses at a large, tertiary care children’s hospital. A large proportion of patients underwent I&D based on history and physical examination alone. However, the use of adjunct studies such as laboratory evaluation and imaging persisted without a demonstrable impact on management or outcomes.

The diagnosis of subcutaneous abscesses was made on history and physical exam alone in approximately 40% of patients in our study population. The fluctuance determined on exam was significantly associated with a need for I&D. However, an ultrasound study was obtained in a large proportion of patients with documented fluctuance. Our data indicate that ultrasound may not be necessary in patients with clearly defined fluctuance on exam and should be reserved for patients with indeterminate exams. Notably, three children in our study underwent I&D after positive ultrasound with no evidence of fluctuance on physical exam, highlighting the role of ultrasound when the diagnosis is uncertain. These findings are similar to the current literature which only recommends ultrasound evaluation when the diagnosis of abscess is equivocal [4,12,13,14].

Laboratory evaluation was performed in a large number of patients, and while an increased WBC count and CRP were not associated with a need for I&D or recurrence, they were associated with a need for inpatient admission. However, other variables such as the heart rate, respiratory rate, and the area of the abscess on exam were also associated with a need for inpatient admission, suggesting minimal benefit to routine laboratory evaluation. Only 13.6% of all wound cultures ordered resulted in a change in antibiotic management. Our data support the previous clinical studies that reject the need for blood cultures in most cases of pediatric subcutaneous abscesses [12,15] and suggest that the role of cultures in simple SSTIs must be reconsidered.

I&D is the treatment of choice for an abscess [6], and the current consensus recommends the routine use of antibiotics after incision and drainage [6,7,9,16]. While we found that IV antibiotics were associated with decreased recurrence, our study found no difference in recurrence based on the use of an oral antibiotic regimen. In addition to I&D, most wounds were either packed with gauze or were drained using a vessel loop; however, neither packing nor loop drain placement were associated with decreased recurrence.

There are several limitations to this study due to the retrospective nature of the study design. It is difficult to determine whether or not certain exam findings were documented before or after obtaining imaging results, such as fluctuance seen on ultrasound, or why certain tests were ordered for various patients. In addition, some children were treated with oral antibiotics prior to presentation, which could be a potential confounder. However, additional analysis revealed no significant difference in evaluation, management, or long-term outcomes between groups. An important limitation is the sample size. While it was adequately powered (*N* = 200) to identify several significant trends, there was great variability in the documentation and diagnostic tests ordered, making the sample size for each individual variable fairly small. Additionally, our knowledge of recurrence was limited by the inability to note a follow-up or recurrence if the patient did not return to a provider within our institution’s healthcare network. Future prospective studies are needed to fully elucidate the optimal diagnosis and management of subcutaneous abscesses.

In conclusion, the incidence of subcutaneous abscesses continues to rise, especially among the pediatric population. However, a significant variability in diagnosis and management across and within institutions remains. The diagnosis of subcutaneous abscess remains a clinical diagnosis that can be made based on history and physical examination alone in the majority of patients. Additional laboratory, microbiology, and imaging studies should be reserved for indeterminate cases and are superfluous in the management of routine subcutaneous abscesses. Future studies are needed to further evaluate and develop a protocolized approach to the diagnosis and management of pediatric subcutaneous abscesses.

## Figures and Tables

**Table 1:** Demographics and patient characteristics.

Variable		All (*n* = 200)	Variable	All (*n* = 200)
Age (years)		4.75 ± 5.4	Surrounding Cellulitis	110 (55.0%)
Gender	Male	88 (44.0%)	Fluctuance	81 (40.5%)
Ethnicity	Hispanic	84 (42.0%)	Multiple Abscesses	29 (14.5%)
BMI		20.1 ± 10.0	Heart rate (beats/minute)	123 ± 26.3
Co-morbidities		11 (5.5%)	Respiratory Rate (breaths/minute)	25.4 ± 5.3
Previous oral antibiotic use		58 (29.0%)	WBC count (10^9^ cells/L)	17.5 ± 6.9
Duration of oral antibiotics		3.97 ± 4.9	CRP level (mg/L)	6.4 ± 5.2
History of MRSA		12 (6.0%)	Area of Abscess (cm^2^)	24.4 ± 34.9
History of prior abscess		60 (30.0%)	Wound Culture obtained	83 (41.5%)
Subjective Fever		101 (50.5%)	Blood Culture obtained	43 (21.5%)

BMI, body mass index; MRSA, methicillin-resistant *Staphylococcus aureus;* WBC, white blood cell; CRP, c-reactive protein; I&D, incision and drainage.

**Table 2:** Diagnostic factors associated with operative incision and drainage.

Variable		All (*n* = 200)	I&D (*n* = 121)	No I&D (*n* = 79)	*p* Value
Exam Findings	Fluctuance	81 (40.5%)	58 (47.9%)	23 (29.1%)	0.005
	Cellulitis	110 (55.0%)	70 (57.9%)	40 (50.6%)	0.07
	Fluctuance + Cellulitis	42 (21.0%)	31 (25.6%)	11 (13.9%)	0.052
Temperature (°C)		37.2 ± 0.8	37.2 ± 0.8	37.2 ± 0.8	0.96
Heart rate (beats/min)		123 ± 26.3	123.0 ± 24.5	123.1 ± 28.6	0.98
Respiratory Rate (breaths/min)		25.4 ± 5.3	25.0 ± 4.9	26.0 ± 5.6	0.22
WBC count (10^9^ cells/L)		17.5 ± 6.9	18.6 ± 6.4	15.7 ± 7.5	0.14
CRP level (mg/L)		6.4 ± 5.2	6.9 ± 5.8	5.6 ± 3.9	0.53
Area of Abscess (cm^2^)		24.4 ± 34.9	28.8 ± 39.4	17.2 ± 24.5	0.05
Ultrasound Performed		108 (54.0%)	65 (53.7%)	43 (54.4%)	1.0

WBC, white blood cell; CRP, c-reactive protein; I&D, incision and drainage.

**Table 3:** Factors for hospital admissions.

Variable		All (*n* = 200)	Admitted (*n* = 55)	Not Admitted (*n* = 145)	*p* Value
Age (years)		4.75 ± 5.4	3.4 ± 4.5	5.3 ± 5.6	0.02
Gender	Male	88 (44.0%)	21 (38.2%)	67 (46.2%)	
Ethnicity	Hispanic	84 (42.0%)	16 (29.1%)	68 (53.1%)	
BMI		20.1 ± 10.0	20.5 ± 11.8	19.5 ± 6.7	0.68
Co-morbidities		11 (5.5%)	5 (9.1%)	6 (4.1%)	
Previous oral antibiotic use		58 (29.0%)	20 (36.4 %)	38 (26.2%)	
History of MRSA		12 (6.0%)	5 (9.1%)	7 (4.8%)	
History of prior abscess		60 (30.0%)	14 (25.5%)	46 (31.7%)	
Subjective Fever		101 (50.5%)	40 (72.7%)	61 (42.1%)	
Surrounding Cellulitis		110 (55.0%)	46 (83.6%)	64 (44.1%)	
Fluctuance		81 (40.5%)	27 (49.1%)	54 (37.2.0%)	
Multiple Abscesses		29 (14.5%)	11 (20.0%)	18 (9.0%)	
Temperature (°C)		37.2 ± 0.8	37.1 ± 0.7	37.3 ± 1.0	0.14
Heart rate (beats/minute)		123 ± 26.3	132.5 ± 23.2	119.5 ± 26.4	0.002
Respiratory Rate (breaths/minute)		25.4 ± 5.3	27.5 ± 6.0	24.6 ± 4.7	0.001
WBC count (10^9^ cells/L)		17.5 ± 6.9	19.1 ± 6.9	14.2 ± 5.8	0.01
CRP level (mg/L)		6.4 ± 5.2	8.0 ± 5.2	3.8 ± 4.0	0.04
Area of Abscess (cm^2^)		24.4 ± 34.9	47.8 ± 50.5	16.2 ± 22.7	0.001
Ultrasound performed		108 (54.0%)	36 (65.4%)	72 (49.7%)	
I&D performed		121 (60.5%)	37 (67.3%)	84 (57.9%)	
Wound Culture		83 (41.5%)	41 (74.5%)	42 (20.0%)	
Blood Culture		43 (21.5%)	33 (60.0%)	10 (6.9%)	

BMI, body mass index; MRSA, methicillin-resistant *Staphylococcus aureus;* WBC, white blood cell; CRP, c-reactive protein; I&D, incision and drainage.

**Table 4:** Factors associated with abscess recurrence within 30 days.

Variable	Recurrence (*n* = 16)	No Recurrence (*n* = 184)	*p* Value
I&D	12 (75.0%)	42 (22.3%)	0.17
IV antibiotics	5 (31.3%)	51 (27.7%)	0.005
Oral antibiotics	4 (25.0%)	23 (12.5%)	0.63
Wound packed	2 (12.5%)	22 (12.0%)	0.29
Loop drain placed	1 (6.3%)	15 (8.2%)	0.09
Temperature (°C)	37.4 ± 1.17	37.2 ± 0.83	0.30
Heart rate (beats/minute)	122.7 ± 34.1	126.8 ± 24.1	0.58
Respiratory Rate (breaths/minute)	24.6 ± 5.4	26.3 ± 6.0	0.29
WBC count (10^9^ cells/L)	13.9 ± 6.0	18.8 ± 7.0	0.15
CRP level (mg/L)	2.14 ± 2.9	8.0 ± 5.5	0.17
Area of Abscess (cm^2^)	14.3 ± 15.5	40.0 ± 48.4	0.08

WBC, white blood cell; CRP, c-reactive protein; I&D, incision and drainage.

## Data Availability

The data presented in this study are available on request from the corresponding author. The data are not publicly available due to privacy.

## Supplementary Materials

The following are available online at https://www.mdpi.com/article/10.3390/children8050392/s1, Table S1: Demographics and patient characteristics of patients without previous oral antibiotic use.
