# Supplementary material for: Pediatric Subcutaneous Abscess: Still a Clinical Exam-Based Diagnosis and Treatment
Source: Children (Basel). 2021 May 14;8(5):392. doi: 10.3390/children8050392 (PMC8153592; doi:10.3390/children8050392)
Supplement: Supplementary file 1 [file children-08-00392-s001.zip › children-1202184-supplementary.pdf]

Supplemental Table 1. Demographics and patient characteristics of patients without previous oral antibiotic use.

**Variable**		**All (*n* = 200)**	**Abx (*n* = 58)**	**No Abx (*n* = 142)**	***p-value***
Age (years)		4.75 ± 5.4	4.36 ± 5.8	4.91 ± 5.2	0.52
Gender	Male	88 (44.0%)	27 (46.6%)	61 (43.0%)	0.64
Ethnicity	Hispanic	84 (42.0%)	20 (34.5%)	64 (45.1%)	0.21
BMI		20.1 ± 10.0	20.7 ± 7.5	19.7 ± 11.2	0.67
Co-morbidities		11 (5.5%)	1 (1.7%)	10 (7.0%)	0.18
History of MRSA		12 (6.0%)	4 (6.9%)	8 (5.6%)	0.75
History of prior abscess		60 (30.0%)	18 (31.0%)	42 (29.6%)	0.23
Subjective Fever		101 (50.5%)	32 (55.2%)	69 (48.6%)	0.44
Exam Findings	Fluctuance	54 (38.3%)	27 (46.6%)	54 (38.0%)	0.16
	Cellulitis	110 (55.0%)	36 (62.1%)	74 (52.1%)	0.32
	Both	42 (21.0%)	14 (24.1%)	28 (19.7%)	0.57
Temperature (°C)		37.2 ± 0.8	37.2 ±0.74	37.2 ± 0.81	0.58
Heartrate (beats/minute)		123 ± 26.3	125.5 ± 23.6	122.0 ± 27.1	0.57
Respiratory Rate (breaths/minute)		25.4 ± 5.3	25.7 ± 4.4	25.3 ± 5.6	0.66
WBC count (10^9 cells/L)		17.5 ± 6.9	18.9 ± 6.8	17.0 ± 6.9	0.33
CRP level (mg/L)		6.4 ± 5.2	8.2 ± 6.5	5.6 ± 4.2	0.21
Area of Abscess (cm^2)		24.4 ± 34.9	31.5 ± 45.1	21.9 ± 30.2	0.15
Ultrasound Performed		108 (54.0%)	38 (65.5%)	70 (49.3%)	0.04
I&D Performed		121 (60.5%)	34 (58.6%)	87 (61.3%)	0.75
Wound Culture obtained		83 (41.5%)	28 (48.3%)	55 38.7%)	0.27
Blood Culture obtained		43 (21.5%)	11 (19.0%)	32 (22.5%)	0.71
Packing		36 (18.0%)	10 (17.2%)	26 (18.3%)	1.0
Drain placement		17 (8.5%)	5 (8.6%)	12 (8.5%)	1.0
IV Antibiotics		70 (35.0%)	27 (46.6%)	43 (30.3%)	0.03
Admitted		55 (27.5%)	20 (34.5%)	35 (24.6%)	0.17
Recurrence		25 (12.5%)	8 (13.8%)	17 (12.0%)	1.0

BMI, body mass index; MRSA, methicillin-resistant *Staphylococcus aureus;.*
WBC, white blood cell; CRP, c-reactive protein; I&D, incision and drainage.
